# Sources and culture methods for the isolation and cultivation of *Coxiella burnetii*

**DOI:** 10.3389/fmicb.2026.1937451

**Published:** 2026-09-23

**Authors:** Jiwon Kim, Ik-Jun Park, Mi-Na Han, Jonghyun Kim, Sanghee Park, Sezim Monoldorova, Bo-Young Jeon, Hye-Won Jeong, Sungsu Youk

**Affiliations:** 1 Microbiology Laboratory, Department of Medicine, College of Medicine, Chungbuk National University, Cheongju, Republic of Korea; 2 Institute of Chungbuk Provincial Veterinary Service and Research, Cheongju, Republic of Korea; 3 Division of Zoonotic and Vector-Borne Disease Research, Center for Infectious Diseases Research, Korea National Institute of Health, Cheongju, Republic of Korea; 4 Department of Biomedical Laboratory Science, College of Health Science, Yonsei University, Wonju, Republic of Korea; 5 Department of Internal Medicine, Chungbuk National University Hospital, Cheongju, Republic of Korea; 6 Biomedical Research Institute, Chungbuk National University Hospital, Cheongju, Republic of Korea

**Keywords:** *Coxiella burnetii*, cultivation, culture methods, isolation, laboratory methods

## Abstract

*Coxiella burnetii*, the bacterium responsible for Q fever, is a highly infectious, environmentally resilient, and aerosol-transmissible zoonotic pathogen. While molecular and serological assays are the primary tools for routine diagnosis, isolating viable *C. burnetii* remains crucial for comprehensive pathogen characterization, including whole-genome sequencing, phenotypic analysis, antimicrobial susceptibility testing, vaccine development, and diagnostic assay validation. This review examines the literature on two key factors for successful *C. burnetii* recovery: selecting an appropriate specimen source and choosing a compatible cultivation platform. Viable organisms can be recovered from human clinical specimens, such as acute-phase blood and tissues from focal chronic infections (e.g., cardiac valves), provided that bacterial burden and viability are maintained. Animal-derived specimens, especially reproductive tissues, placental material, fetal tissues, milk, urine, feces, and genital secretions, are vital sources for veterinary investigations and zoonotic risk assessment. However, contamination with other microorganisms often complicates recovery from these samples. Tick-derived and environmental samples can broaden epidemiological insights, but their interpretation requires caution, as Coxiella-like endosymbionts and low viable bacterial burdens may hinder culture recovery. Regarding culture platforms, mammalian cell culture, particularly shell vial-based systems, is the most widely applicable method for primary isolation. Embryonated chicken eggs and animal inoculation are still valuable for biological amplification or generating phase variants. Furthermore, host cell-free axenic media have revolutionized *C. burnetii* propagation, metabolic analysis, and genetic studies. Successful isolation hinges on several factors: specimen type, processing, storage conditions, bacterial viability, strain characteristics, and compatibility with the chosen culture system. Future advancements should incorporate viability-based molecular preselection, high-throughput image-based culture screening, optimized cell-line selection, axenic media with broader strain permissiveness, and standardized reporting of culture attempts. These improvements will expand available isolate collections and diversify studies on *C. burnetii* biology, epidemiology, and control.

## 1 Introduction

Q fever, caused by *Coxiella burnetii* (*C. burnetii*), is a globally distributed zoonosis with public health implications. *C. burnetii* is highly infectious, environmentally resilient, and transmitted via aerosols. Its ability to survive in the environment for extended periods facilitates sustained transmission from animal reservoirs to humans. These characteristics, combined with its low infectious dose, efficient aerosol dispersal, and stability during storage and dissemination, have led to its recognition as a potential biological threat agent.

From a biosafety and biosecurity standpoint, *C. burnetii* necessitates stringent laboratory controls. It is classified as a Risk Group 3 pathogen in numerous countries, including the United States and European Union member countries, with similar classifications globally. In the United States, it is also designated a Select Agent, and in the European Union, it is subject to dual-use oversight. Multiple laboratory-acquired infections have been documented, particularly during the handling or processing of specimens or cultures with high bacterial loads (Johnson and Kadull, 1966; Curet and Paust, 1972; Hall et al., 1982; Graham et al., 1989; Wurtz et al., 2016). Consequently, laboratory procedures involving viable *C. burnetii* or specimens suspected of containing high bacterial loads must be performed under Biosafety Level 3 (BSL-3) or higher containment to minimize laboratory-acquired infections and environmental release, irrespective of regulatory exemption status. For instance, while attenuated strains like Nine Mile (NM) phase II may be exempt from Select Agent regulations under specific conditions, a thorough biosafety risk assessment remains crucial. This is because this strain can undergo lipopolysaccharide elongation through genetic reversion, leading to phenotypic changes associated with increased virulence (Long et al., 2024; Pierce et al., 2026).

The successful isolation of *C. burnetii* is influenced by sample type, specimen quality, storage conditions, bacterial viability, and pathogen burden. PCR positivity alone does not guarantee culture recovery, as molecular assays can detect DNA from nonviable organisms or residual bacterial material. Even when viable bacteria are present, isolation can be inefficient, especially for wild-type phase I *C. burnetii*, which is generally more challenging to establish in cell culture than phase II strains (Omsland et al., 2011). Furthermore, strain-specific differences can impact infectivity and propagation in commonly used culture systems. Therefore, culture-based detection is not suitable for routine Q fever diagnosis; PCR and serology remain the primary diagnostic approaches (Henning and Sting, 2002; Niemczuk et al., 2014).

Despite the technical demands, time commitment, and reliance on high-containment facilities, *C. burnetii* culture is indispensable for comparative biology, pathogenesis studies, antimicrobial testing, vaccine development, and diagnostic assay validation. This review summarizes published methods for *C. burnetii* isolation and culture, with a focus on approaches that enable the recovery and propagation of viable organisms from host-derived matrices, including embryonated eggs, cell culture systems, and host cell-free culture. Animal inoculation is discussed solely in the context of contaminant reduction or selective enrichment, rather than as a standardized isolation method.

## 2 Sources for *C. burnetii* isolation

### 2.1 Human clinical samples

A range of fresh or frozen clinical specimens can serve as sources for *C. burnetii* isolation, including anticoagulated whole blood, serum, cardiac valve tissue, vascular grafts, bone marrow, placenta, and other tissues from acute or chronic Q fever cases. Blood collected during acute bacteremia and tissue from focal chronic infections, such as infected cardiac valves, have been reported as favorable sources for cultivation.

(1) Isolation from tissue, reproductive, and genitourinary specimens

*C. burnetii* has been successfully isolated from cardiac valve tissues obtained at autopsy from patients with infective endocarditis prior to antimicrobial therapy (Raoult et al., 1990; Boden et al., 2015). Notably, *C. burnetii* has also been recovered from valvectomy specimens from patients with chronic Q fever, even after prolonged antibiotic treatment. This demonstrates the organism’s ability to persist within infected tissues despite extended antimicrobial exposure (Muhlemann et al., 1995; Boden et al., 2015; Celebi et al., 2019). Less commonly, isolation has been achieved from other tissue or fluid specimens, such as bone biopsy, spleen biopsy, and synovial fluid, using shell vial culture-based cell culture systems (Santos et al., 2012; Melenotte et al., 2018).

Reproductive and genitourinary specimens may also be useful in selected clinical contexts. In one case involving spontaneous abortion, *C. burnetii* was isolated using the yolk sac inoculation method from placental fragments, genital and fecal swabs, and urine samples stored at −20 °C (Vaidya et al., 2008). Urine and semen collected during the acute phase of infection have also yielded positive results after inoculation onto susceptible cell cultures, indicating that viable organisms may occasionally be recovered from non-blood clinical specimens during acute Q fever (Kruszewska et al., 1996).

(2) Isolation from blood-derived specimens

Anticoagulated whole blood, plasma, buffy coat fractions, serum, and peripheral blood mononuclear cells have served as blood-derived sources for isolating *C. burnetii*. While anticoagulated whole blood has been deemed suitable for animal-based isolation (Oda and Yoshiie, 1989), plasma/buffy coat fractions, obtained after whole blood sedimentation, are typically preferred for inoculation into cell lines, embryonated eggs, or immunosuppressed mice (Paretsky et al., 1958; Spyridaki et al., 1998; Gouriet et al., 2005). The choice of anticoagulant may be worth considering as a factor that could influence isolation efficiency. Notably, EDTA- or citrate-anticoagulated blood remains preferred for molecular detection (Anderson et al., 2013; Mori et al., 2017). However, chelation of Mg^2+^ and Ca^2+^ has been shown to destabilize the lipopolysaccharide-rich outer leaflet of Gram-negative bacteria (Vaara, 1992; Nikaido, 2003). Consistent with this, heparinized blood has been used for the isolation of *C. burnetii* (Musso and Raoult, 1995; Gouriet et al., 2005). Heparinization therefore appears to be a reasonable starting point when isolation is planned in advance. The anticoagulants have not been compared directly for *C. burnetii*, however, and evaluation would be needed to confirm this.

Serum samples from acute-phase Q fever patients have also been used to inoculate cell lines (Nagaoka et al., 1996; Santos et al., 2009; Vincent et al., 2015; Graves and Islam, 2016; Lee et al., 2020). However, these studies did not specify whether serum was collected in gel-barrier tubes. This detail may be relevant because gel-barrier serum tubes usually require moderate-speed centrifugation for serum separation, which exceeds the low-speed centrifugation typically employed in shell vial culture. Such processing might reduce the number of bacteria in the inoculum, thereby lowering the probability of isolation.

In summary, blood-derived specimens are useful for *C. burnetii* isolation, but their suitability can vary depending on the anticoagulant, processing method, centrifugation conditions, and the intended cultivation platform.

### 2.2 Animal-derived samples

Animal-derived specimens have been crucial for isolating *C. burnetii* in studies of reproductive disorders, environmental shedding, and zoonotic transmission. Sheep, goats, and cattle are primary animal reservoirs of *C. burnetii*, shedding the organism in various matrices such as milk, urine, feces, genital secretions, and placental tissues. Due to the presence of commensal or contaminating microorganisms in these specimens, sample processing typically involves homogenization, clarification, filtration, and treatment with antibacterial and antifungal agents. Penicillin, streptomycin, and amphotericin B can be added to samples before inoculation or during the early stages of cultivation in embryonated eggs or cell culture systems to suppress unwanted microbial growth (Mori et al., 2017; WOAH, 2018). Specifically, tissue samples are usually homogenized in serum-free medium, clarified through low-speed centrifugation, and then sequentially filtered before being inoculated into shell vial cultures.

(1) Reproductive tissues: placenta and birth fluids

Reproductive tissues and abortion-associated specimens are crucial sources for isolating *C. burnetii* due to the organism’s ability to accumulate to high burdens within them. Placental cotyledons, in particular, are relevant because they are highly vascularized, metabolically active tissue niches that mediate exchange between maternal and fetal compartments.

In sheep, *C. burnetii* has been isolated from placenta and wool, highlighting the importance of parturition-associated materials (Stoker et al., 1955; Morelj et al., 1957). While one study found eight samples from aborted sheep fetuses to be *IS1111* PCR-positive, yolk sac inoculation of these material yielded only a single isolate (Kilicoglu et al., 2020). Other studies have reported successful isolation or attempts from aborted fetal liver samples from cattle, sheep, and goats (Santos et al., 2012), sheep fetal liver and goat fetal specimens (Cumbassá et al., 2015), and placenta, internal organs of aborted fetuses, and vaginal swabs from ruminants, including goats, cattle, and sheep (Niemczuk et al., 2014).

Cattle reproductive disease-associated samples have also provided significant isolation sources. *C. burnetii* was isolated via mouse passage from uterine swabs, fetal tissues, milk, and mammary gland samples collected from cattle with reproductive disorders (Ho et al., 1995). In another investigation, milk, feces, and genital swabs from cattle with a history of reproductive disorders were processed and inoculated into embryonated eggs, with infection subsequently confirmed using yolk sac membrane smears (Vaidya et al., 2010). Additionally, successful isolation has been achieved by inoculating embryonated eggs with blood samples from cattle and buffaloes experiencing recurrent abortions (Das et al., 2014).

Beyond domestic ruminants, feline reproductive specimens have also been implicated. The first reported *C. burnetii* isolation from cats came from vaginal swabs collected near the uterine cervix of female cats (Nagaoka et al., 1998). Cats with outdoor access have also been recognized as potential sources of *C. burnetii* infection (Egberink et al., 2013). Historical reports further describe isolation from wild shrew mice (Syrucek et al., 1957), suggesting that non-ruminant mammals may also serve as potential sources for *C. burnetii* isolation.

(2) Secretions and excretions: milk, urine, feces, and related samples

*C. burnetii* has been detected and/or isolated from the milk, urine, feces, and genital secretions of infected ruminants. Early studies reported isolating the organism from sheep urine and goat milk (Morozzi, 1951; Likar, 1955). These findings suggest that viable organisms can be shed through non-tissue matrices, although isolation is generally more successful from reproductive or abortion-associated tissues.

Raw milk requires preprocessing because its fat content can interfere with inoculation and subsequent culture. Simple decantation has been used to “decream” milk, with the material aspirated from beneath the fat layer serving as the inoculum. This subcream fraction is likely advantageous because it reduces the inhibitory effect of excess fat while retaining somatic cells and cell-associated organisms that may contain viable *C. burnetii*. Several studies have successfully used milk or milk-associated preparations for isolation. For instance, in a study of unpasteurized commercially available cow’s milk, organisms were concentrated from raw milk, mildly heat-treated to reduce bacterial contaminants, and then evaluated by mouse passage and RK13 cell culture (Loftis et al., 2010). In another study, milk and other cattle-derived samples were diluted in sucrose-phosphate-glutamate buffer and inoculated directly into mice with minimal additional preparation (Ho et al., 1995). These reports confirm that raw milk can be a source for *C. burnetii* isolation.

Fecal and genital specimens have also yielded *C. burnetii* isolates, including samples from cattle with reproductive disorders and vaginal swabs from cats (Ho et al., 1995; Nagaoka et al., 1998; Vaidya et al., 2010). While these findings indicate that secretions and excretions can serve as isolation sources, they are generally more challenging than tissue specimens due to lower bacterial loads and a higher risk of contamination.

(3) Clinical fluid samples: blood and serum

Blood and serum from animals have also been used to isolate *C. burnetii*, particularly in cases associated with abortion, reproductive disorders, or suspected zoonotic transmission. In cattle, blood samples have been targeted in areas with reported human Q fever, including studies of artificial-insemination semen storage facilities (Kruszewska and Tylewska-Wierzbanowska, 1997). Blood samples from cattle and buffaloes with histories of recurrent abortion have also been inoculated into embryonated eggs, resulting in successful isolation (Das et al., 2014). These findings show that *C. burnetii* can be isolated from animal blood samples, particularly during active infection or reproductive disease, and underscore the epidemiological value of animal blood sampling for tracing potential sources of human infection.

### 2.3 Tick-derived samples

Tick-derived samples bridge the gap between animal reservoirs and environmental surveillance, offering insights into both *C. burnetii* viability and vector/reservoir ecology. However, their interpretation requires careful consideration. *C. burnetii* has been detected in various tick species (Eldin et al., 2017; Korner et al., 2021) and can persist in tick tissues, primarily through transstadial transmission (Smith, 1941). It may be released in saliva or feces during blood feeding (Korner et al., 2020). However, PCR detection alone is insufficient to confirm *C. burnetii* due to the presence of Coxiella-like endosymbionts in ticks. For this reason, confirmatory PCR targeting *C. burnetii*-specific genes, such as *com1* and *htpAB* is preferred in addition to Coxiella-wide *IS1111* PCR (Mathews et al., 2024; Tolpinrud et al., 2024). However, *com1* and *htpAB* are single-copy genes, so their sensitivity is lower than that of multicopy *IS1111*. Therefore, a two-step approach is preferable, in which *IS1111*-positive samples are subsequently verified using single-copy targets and sequencing rather than relying on detection with a single target alone (Korner et al., 2021).

The NM strain, a representative tick-derived isolate, was recovered from *Dermacentor andersonii* in Montana. This was achieved through initial guinea pig inoculation, followed by propagation in embryonated eggs (Davis and Cox, 1938). Subsequently, Smith (1941) provided one of the earliest experimental demonstrations of *C. burnetii* recovery from infected *Rhipicephalus sanguineus* ticks by inoculating susceptible guinea pigs (Smith, 1941). Later studies reported *C. burnetii* isolation from ticks associated with infected or epidemiologically linked animals. These included *Rhipicephalus sanguineus* from an infected dog (Mantovani and Benazzi, 1953), *Ixodes holocyclus* from cattle linked to a Q fever case (Carley and Pope, 1953), *Amblyomma triguttatum* from seropositive kangaroos (Pope et al., 1960), and *Ixodes* spp. from dairy cattle with reproductive disorders (Ho et al., 1995).

Large-scale environmental tick surveys have further supported ticks as potential isolation sources. In Slovakia, approximately 7,000 ticks were examined, leading to the isolation of *C. burnetii* by yolk sac inoculation from seven species across the genera *Dermacentor*, *Ixodes*, and *Haemaphysalis* (Rehacek et al., 1991). *C. burnetii* was also recovered by Vero shell vial culture from *Hyalomma* spp. hemolymph and homogenized *Rhipicephalus sanguineus* collected from goat and sheep farms (Spyridaki et al., 2002). In contrast, despite *IS1111* PCR detection *C. burnetii* DNA in several tick taxa collected from cattle, sheep, and goats, including *Hyalomma*, *Boophilus*, and *Haemaphysalis* spp., yolk sac inoculation of PCR-positive samples failed to yield isolates (Kilicoglu et al., 2020). Overall, while tick samples are valid sources for *C. burnetii* isolation, recovery success varies depending on tick species, sample types, and culture platforms.

### 2.4 Environmental samples

Environmental sampling is useful for assessing *C. burnetii* contamination in ruminant livestock settings, particularly on sheep, goat, and cattle farms experiencing clinical or reproductive disease, and in human-activity areas where background environmental contamination requires evaluation. Among environmental matrices, air and soil sampling are particularly suited to assessing aerosol transmission and environmental persistence, respectively (Kersh et al., 2010; Abeykoon et al., 2021). Farm dust appears to be an informative matrix, with higher detection rates reported on sheep and mixed ovine-caprine farms (Zendoia et al., 2021; García-Pérez et al., 2025). Airborne dust in a sheep barn was also shown to contain *C. burnetii* during the shearing of a seropositive herd, supporting the relevance of aerosol-associated environmental sampling (Schulz et al., 2005).

Although environmental sampling is primarily used for molecular detection, successful isolation from environmental sources has occasionally been reported. Stable dust was the first documented environmental source from which *C. burnetii* was isolated (Vuksic et al., 1956). Isolation has also been reported from old barn hay linked to an acute Q fever case after exposure during hay cleaning. Because such materials are often heavily contaminated, antimicrobial pretreatment with amphotericin B and gentamicin was used to suppress background microorganisms (Rustscheff et al., 2000). Overall, farm-associated environmental samples are valuable for detecting *C. burnetii*. However, they are generally less favorable for culture than high-burden clinical or reproductive tissues due to low viable bacterial loads, degradation, inhibitory substances, and competing microbial contamination.

## 3 Specimen selection and storage for *C. burnetii* isolation

The success of isolating *C. burnetii* is primarily determined by the bacterial load. Vincent et al. (2015) attempted isolation from 64 acute-phase serum samples from confirmed acute Q fever patients. They defined molecular positivity as a cycle threshold (Ct) ≤ 40 in both *com1* and *htpAB* qPCR assays or Ct ≤ 35 in *com1* alone (Vincent et al., 2015). However, *C. burnetii* was recovered after cell inoculation even from serum initially negative by *com1* qPCR, suggesting that viable organisms can persist below the detection limit. Nevertheless, since qPCR Ct values generally correlate with bacterial burden, prioritizing samples based on Ct values can be useful when considering a large number of specimens for isolation attempts. This aligns with our experience, in which samples with lower Ct values were more likely to yield successful *C. burnetii* isolation.

Storage stability varies by specimen type. In whole blood and blood fractions, *C. burnetii* viability was maintained for up to 6 weeks at 1–6 °C (Kersh et al., 2013). For serum, successful recovery was reported from samples refrigerated for up to 224 days or frozen at −20 °C for up to 371 days (Vincent et al., 2015). In heart valve tissue, isolation was achieved even after several years of storage at −25 °C (Boden et al., 2015). These observations indicate that *C. burnetii* can remain recoverable from various clinical matrices after prolonged storage, supporting the feasibility of retrospective isolation studies using archived sample banks.

## 4 Experimental animal inoculation for contaminant reduction and enrichment

Experimental animal inoculation initially served as primary technique for isolating *C. burnetii*. Since the establishment of cell culture method, it has instead served as auxiliary method, especially when original specimens are likely to have significant bacterial or fungal contamination. This approach also helps maintain the native virulent phase I phenotype of *C. burnetii*, which can be less stable during prolonged *in vitro* passage. However, animal-based isolation is less standardized, more time-consuming, and ethically more demanding than methods using cell culture or embryonated eggs. Consequently, it is best considered a supportive method for challenging or contaminated specimens rather than a routine isolation platform.

For isolation purposes, intraperitoneal inoculation is generally preferred over respiratory routes. While respiratory inoculation, whether via intratracheal route or aerosol exposure, more closely mimics natural infection and is valuable for studying pulmonary Q fever pathogenesis (Schoffelen et al., 2015; Priestley et al., 2021), intraperitoneal inoculation in a severe combined immunodeficient (SCID) mouse tissue model resulted in higher initial tissue bacterial burdens than intratracheal inoculation. Nevertheless, lung, liver, and spleen homogenates from both routes supported reisolation in ACCM-2 (Omsland et al., 2011). This difference may be attributed to *C. burnetii*’s ability to replicate efficiently within mouse peritoneal macrophages and achieve high titers (Zamboni et al., 2002).

### 4.1 Guinea pigs

Early investigations demonstrated that guinea pigs readily developed infection after inoculation with blood or urine from patients, and their subsequent immune responses enabled disease-specific diagnosis (Davis and Cox, 1938; Derrick, 1937). Guinea pigs are also relatively susceptible to *C. burnetii* compared with immunocompetent inbred mouse strains (Tamrakar et al., 2011). The manifestation of clinical signs depends on the inoculation route: intraperitoneal inoculation commonly induces dose-dependent fever and liver-associated pathological changes, whereas intranasal inoculation produces greater pulmonary involvement. These features make guinea pigs valuable for pathogenicity assessment. However, guinea pigs are less practical than mice due to their greater space requirements, higher maintenance costs, and the need for larger inoculum volumes.

### 4.2 Mice

(1) Immunodeficient and immunocompromised mice

Immunodeficient mice have demonstrated experimental susceptibility to *C. burnetii*. Among various mouse models, complement-deficient A/J mice and SCID mice (lacking functional T and B cells) are highly susceptible to infection (Scott et al., 1987; Ho et al., 1995; Nagaoka et al., 1998; Tamrakar et al., 2011). SCID mice exhibit exceptional sensitivity to low-dose infections, supporting persistent bacterial replication, amplification, and the development of chronic lesions, including endocarditis-like pathology (Andoh et al., 2003). Their high sensitivity may facilitate the recovery of *C. burnetii* from low-burden specimens, with reports indicating SCID mice are more sensitive than cell culture for isolating certain strains (Lockhart M. et al., 2012). Nevertheless, due to their impaired adaptive immunity, these animals may be less suitable when the primary objective is to eliminate microbial contaminants from heavily contaminated samples.

Immunosuppressed mice have also been employed to enhance *C. burnetii* isolation from clinical specimens (Sidwell et al., 1964a,b; Kishimoto et al., 1978). In one method, heparinized human whole blood was subcutaneously inoculated into BALB/c mice treated with cyclophosphamide on the day of inoculation and again 5 days later. Spleen cells harvested 2 weeks post-inoculation were serially passaged into additional immunosuppressed mice, with clinical signs of infection, such as ruffled fur, observed after the second passage (Oda and Yoshiie, 1989).

(2) Immunocompetent mouse

Immunocompetent mouse strains exhibit substantial differences in susceptibility to *C. burnetii*. C57BL/6 and CBA mice, commonly used in immunological studies, typically do not show mortality or overt clinical disease. While BALB/c mice usually survive, they may develop clinical signs such as lethargy, rough or ruffled fur, reduced movement, and body weight loss (Zhang et al., 2007; Hu et al., 2019). Splenomegaly and hepatomegaly are common findings across susceptible mouse models. The success of *C. burnetii* enrichment through mouse inoculation depends on several factors, including mouse strain, age, inoculation route, infectious dose, and the biological properties of the *C. burnetii* strain. For instance, infection in BALB/cJ mice can be self-limiting without obvious clinical signs. Conversely, C57BL/6J mice have, in some settings, been reported to develop stronger clinical signs, tissue injury, and immune responses than BALB/cJ mice (Baumgärtner et al., 1993). Mouse age also appears to influence the host response to infection (Leone et al., 2007).

### 4.3 Practical use for contaminated specimens

Mouse inoculation is best suited for specimens suspected to contain microbial contaminants or low numbers of viable *C. burnetii*. These specimens can be inoculated intraperitoneally into adult mice older than 50 days or into cyclophosphamide-treated mice. If sufficient material is available and the viable bacterial load is expected to be low, inoculating up to three times at 5- to 7-day intervals may increase the likelihood of establishing an infection. Inoculated animals are typically monitored for clinical signs and evaluated by serology or postmortem examination for approximately 3–5 weeks post-infection. Successful infection is often indicated by splenomegaly, which reflects the *in vivo* propagation of *C. burnetii*. The spleen, liver, and lung are the preferred organs for subsequent recovery of the bacterium and can be used for further passage in embryonated chicken eggs, susceptible cell lines, or host cell-free culture systems.

## 5 Embryonated chicken egg inoculation

Embryonated chicken eggs (ECEs) were among the earliest biological platforms used to isolate, maintain, and propagate *C. burnetii* and other rickettsial agents before cell culture became established. The initial use of ECEs for *C. burnetii* cultivation was integral to the first experimental characterization of Q fever. As early reports from 1937 describe, the D6 strain, originally maintained through serial passage in guinea pigs, was inoculated into ECEs using liver homogenates (Burnet and Freeman, 1937; Derrick, 1937). Early inoculations were performed via the chorioallantoic route, similar to conventional methods for viruses. Subsequent studies, however, revealed that replication in ECEs primarily occurred in the yolk sac rather than embryonic tissues. This led to the adoption of yolk sac inoculation as the standard ECE-based propagation method (Ormsbee, 1952). Thereafter, yolk sac culture was employed for isolation from diverse sources, including sheep placenta, tick-derived material, and rodent samples (Stoker et al., 1955; Stoker and Marmion, 1955; Stoenner and Lackman, 1960; Burgdorfer et al., 1963).

In the conventional ECE method, emulsified or clarified specimens are inoculated into the yolk sac of 5- to 7-day-old embryonated eggs. Growth kinetics in ECEs depend on the inoculum dose and bacterial adaptation. For high-dose inocula of well-established laboratory strains, exponential growth typically occurs approximately 4-to-7 days post-inoculation (Ormsbee, 1952). Therefore, embryonic death before this period is unlikely to be caused by *C. burnetii* multiplication. Since *C. burnetii* replication relies on living host-cell metabolism, bacterial multiplication is expected to cease after embryonic death. Death during the exponential growth phase can thus limit maximal bacterial yield if harvesting is delayed or mistimed. Although *C. burnetii* organisms in dead embryos retained complement-fixation activity, suggesting the persistence of antigenically intact and viable bacteria, this observation was primarily based on studies using the well-adapted NM strain (Ormsbee, 1952). For primary isolation of wild-type strains, longer incubation periods until 10–12 days post-inoculation have been used or recommended as a safer and more practical approach (WOAH, 2018). However, because prolonged incubation may reduce the obtainable yolk sac material, harvest timing should be optimized to maximize bacterial amplification.

ECE inoculation remains historically and technically significant for generating LPS phase variants. A notable example is the derivation of the NM phase II strain from NM phase I, which required 50 serial passages in ECEs (Stoker and Fiset, 1956). Similarly, phase variation of the Priscilla strain necessitated over 30 passages, with a complete transition to a phase II LPS profile observed only after approximately 80 ECE passages (Lukacova et al., 1993; Quevedo Diaz and Lukacova, 1998). While serial passage in cell culture or axenic medium has been explored, these systems have not consistently reproduced the fully truncated phase II LPS profile seen in egg-passaged NM II without intermediate-length LPS forms (Hotta et al., 2002; Kersh et al., 2011). Furthermore, axenic passage studies indicate that the pattern and extent of phase transition can vary among strains (Beare et al., 2018). Consequently, despite its less frequent use in routine isolation, ECE passage remains valuable for the biological amplification, long-term maintenance, and experimental generation of *C. burnetii* phase-transition variants.

## 6 Mammalian cell culture for *C. burnetii* isolation and cultivation

Mammalian cell culture is now a primary method for isolating and propagating *C. burnetii*. The shell vial technique is frequently used for isolating *C. burnetii* and other intracellular bacteria. This method includes centrifugation-assisted inoculation, which improves bacterial contact with the cell monolayer, thereby enhancing attachment and entry. For instance, centrifugation at 500–700 × g for 30–60 min at room temperature is widely accepted. Similar centrifugation protocols have also been applied in other culture formats, such as flasks or microplates (Raoult et al., 1990; Gouriet et al., 2005; Vincent et al., 2015; Francis et al., 2020). Early cell culture studies using Vero and L929 cells revealed pleomorphic bacteria within large cytoplasmic vacuoles (Burton et al., 1978). In L929 cells, long-term infection was linked to flattened rough endoplasmic reticulum and decreased lysosomal activity, indicating host-cell adaptation during persistent infection. Since then, various mammalian cell lines, including macrophage-like, fibroblast, and epithelial cells, have been shown to support *C. burnetii* replication.

### 6.1 Cell lines for isolation

Macrophage-lineage cells are frequently used for *C. burnetii* isolation because macrophages are a primary target during natural infection. Mouse macrophage-like tumor cell lines, such as J774 and P388D1, support *C. burnetii* replication (Baca et al., 1981). The canine malignant histiocytic macrophage cell line DH82 has also been increasingly adopted for *C. burnetii* culture. This is partly due to its established use with other intracellular bacteria that target mononuclear leukocytes, such as *Ehrlichia* spp. DH82 cells have successfully supported the isolation and propagation of multiple MST types, including MST4, MST8, MST13, and MST20 (Santos et al., 2012; Cumbassá et al., 2015). However, strain-dependent susceptibility exists; for instance, DH82 cells were most sensitive to the Henzerling isolate, while Vero cells were more sensitive to the Arandale isolate (Lockhart M. G. et al., 2012). Other macrophage-related systems, including HL-60 and M-CSF-differentiated human monocyte-derived macrophages, have been employed as more physiologically relevant infection models (Shannon and Heinzen, 2008; Santos et al., 2009). Consistent with host-dependent cell susceptibility, bovine macrophages supported faster replication of bovine-derived isolates than goat-derived isolates. This suggests that host species-matched cell models may be beneficial for specific isolation or infection studies (Mori et al., 2013).

Fibroblast cell lines are widely used for *C. burnetii* isolation and long-term propagation. Following the early use of L929 cells, human embryonic lung fibroblast cell lines, such including HEL and MRC-5, were adopted for primary isolation. HEL cells are particularly well-established in shell vial protocols because their contact inhibition properties allow for stable monolayer maintenance during extended incubation. Diagnostic studies have shown efficient recovery of *C. burnetii* in HEL shell vial cultures, with detection often occurring within 10–20 days post-inoculation (Raoult et al., 1990; Gouriet et al., 2005). MRC-5 cells have also been adapted for microplate-based high-content screening systems, enabling automated detection of infection-associated vacuoles (Francis et al., 2020).

Epithelial cell lines offer robust and practical systems for prolonged culture. Vero and Vero E6 cells are commonly used due to their ability to form stable monolayers and tolerate extended incubation (Spyridaki et al., 2002). Vero cells have successfully supported isolation from acute-phase serum, including long-term culture for up to 12 weeks with sequential PCR monitoring of supernatants (Vincent et al., 2015). BGM cells have also been utilized for isolation from environmental samples (Rustscheff et al., 2000). Two human placental cell lines, BeWo and JEG-3, have demonstrated susceptibility to human placental isolates. However, it remains unestablished whether these cells offer advantages for primary isolation compared to conventional models (Zarza et al., 2023).

### 6.2 Culture conditions and detection strategies

Roswell Park Memorial Institute-1640 has been widely used more than minimum essential medium (MEM) for *C. burnetii* inoculation and maintenance (Coleman et al., 2004; Sandoz et al., 2016; Sanchez et al., 2021). This is likely due to its composition, which includes non-essential amino acids, richer vitamins, metabolic cofactors, and relatively high phosphate concentrations that improve buffering capacity and pH stability. While MEM supplemented with fetal calf serum and L-glutamine has also been used in HEL shell vial cultures, the optimal medium choice should align with the specific cell line, inoculum type, and anticipated incubation period.

Traditionally, *C. burnetii* infection has been confirmed using Gimenez staining, immunofluorescence, and PCR. More recently, automated high-content imaging has emerged as an alternative screening strategy. This method involves maintaining MRC-5 or L929 monolayers in 96-well plates, with cycloheximide used to stabilize the monolayer. Infection is then detected through automated imaging of cytopathic changes, particularly vacuole formation (Francis et al., 2020). This high-throughput approach has the potential to streamline screening and isolation from a large number of samples.

### 6.3 Recovery of intracellular bacteria

Because *C. burnetii* replicates within host-cell vacuoles, achieving higher titers may require additional processing to release organisms from infected cells. Reported release methods include sonication, osmotic stress using distilled water, syringe-needle passage, homogenization, and glass bead beating. These methods are typically followed by high-speed centrifugation of the post-nuclear supernatant to pellet the released bacteria (Baca et al., 1993; Veras et al., 1994; Meconi et al., 1998; Beron et al., 2002; Zamboni and Rabinovitch, 2003; Coleman et al., 2004).

### 6.4 Determinants of minimal bacterial input for successful isolation

The initial bacterial input needed for *C. burnetii* isolation lacks a universal threshold due to variations in bacterial phase, physiological state, strain, host-cell type, and culture format. For instance, NM II in Vero cells exhibited an average genome equivalent-to-focus-forming unit (GE/FFU) ratio of approximately 6:1 when measured by FFU assay post-infection (Cockrell et al., 2008). However, this estimate, derived from purified, cell culture-adapted NM II, may not apply to primary specimens or wild-type phase I bacteria.

The GE/FFU ratio varies markedly with bacterial condition. For example, axenically grown organisms demonstrated varying infectivity based on medium composition: 38.77 GE/FFU in ACCM-2, 6.02 GE/FFU in ACCM-D, and 3.92 GE/FFU in glucose-depleted ACCM-D (Sandoz et al., 2016). In contrast, virulent phase I *C. burnetii* may necessitate a substantially higher input, with approximately 300 GE/FFU suggested for productive infection (R. A. Heinzen, unpublished data, cited in Omsland et al., 2011). Therefore, genome copy numbers cannot be directly converted into infectious units without accounting for bacterial viability.

Practically, assuming a 6 GE/FFU infectivity ratio, approximately 1.2 × 10^6^ GE would be required to achieve efficient synchronous infection of a Vero cell monolayer in a shell vial. However, this estimate applies to one-step growth experiments and should not be misconstrued as the minimum inoculum for primary isolation. As Vincent et al. (2015) discuss, even low numbers of viable organisms can be recovered through prolonged incubation or serial passage. Nonetheless, isolating bacteria from low inputs is time-consuming and susceptible to culture failure. This is because uninfected host cells can overgrow, while infected cells may experience cell cycle arrest, thereby reducing the relative abundance and detectability of infected cells.

## 7 Host cell-free culture of *C. burnetii*

Host cell-free culture has revolutionized experimental work with *C. burnetii*, a bacterium long thought to depend on eukaryotic host cells for growth. These systems mimic the intracellular phagolysosome-like niche where *C. burnetii* naturally replicates, specifically by recreating acidic, nutrient-rich, and low-oxygen conditions.

Early attempts in host cell-free culture used phosphate-based buffers supplemented with simple carbon sources and amino acid mixtures under acidic conditions (Burton et al., 1978; Hackstadt and Williams, 1981). While these formulations did not support robust bacterial expansion, they did demonstrate limited extracellular metabolic activation. This included RNA synthesis-dependent protein synthesis and metabolic activity linked to the early transition from the small cell variant (SCV) to the large cell variant (LCV) (Zuerner and Thompson, 1983).

The systematic development of host cell-free cultivation began with the understanding that *C. burnetii* requires extensive exogenous amino acid supplementation. This need aligns with its multiple amino acid auxotrophies and its use of amino acids as carbon sources. A foundational host cell-free formulation, complex *Coxiella* medium (CCM), incorporated high chloride concentrations, citrate buffering, and complex host-like nutrient complementation. Although CCM did not support significant numerical growth, it maintained axenic metabolic activity for over 24 h (Omsland et al., 2008).

Subsequent analysis of metabolic requirements, using expression microarrays, genomic reconstruction, and metabolite profiling, led to the supplementation of CCM with high concentrations of casamino acids and/or L-cysteine. This enhancement enabled approximately 230-fold expansion of the NM phase II strain. Furthermore, reducing oxygen to 2.5% supported an additional approximate 3-log increase in genome equivalents (GE) (Omsland et al., 2009).

These advancements culminated in acidified citrate cysteine medium (ACCM), which facilitated bacterial replication under low-oxygen conditions. Growth was observed at 1%, 2.5%, and 5% oxygen but not at 10%, confirming the critical role of reduced oxygen. ACCM also supported a biphasic developmental cycle similar to that observed in host cells. For the NM phase II strain, an initial inoculum of at least 100 GE/mL was necessary to achieve exponential growth within 1 week. Notably, the maximum yield obtained from 1 mL of ACCM was comparable to that produced in two T25 flasks of Vero cell culture (Seshadri et al., 2003).

ACCM was refined into ACCM-2 by replacing fetal bovine serum with methyl-β-cyclodextrin, a modification inspired by media used for *Helicobacter pylori* and *Bordetella pertussis*. Methyl-β-cyclodextrin acts as a lipophilic molecule carrier and detoxifying agent. Under moderate shaking and 2.5% oxygen, ACCM-2 supported 4- to 5-log growth over 7 days. Notably, NM phase II could be cultured in ACCM-2 from infected SCID mouse tissue homogenates containing as few as 10 GE/mL (Omsland et al., 2011). Later estimates refined the sensitivity of axenic cultivation to approximately 10-100 GE/mL, depending on sample quality (Mori et al., 2017).

The improvements from ACCM to ACCM-2 extended beyond bacterial yield. Comparative analysis revealed that ACCM failed to support proper LCV-to-SCV transition during the stationary phase after 14 days; bacteria remained predominantly in the LCV state and rapidly lost viability. In contrast, ACCM-2 preserved normal developmental transition and survival. Methyl-β-cyclodextrin was proposed to mitigate L-cysteine-derived oxidative stress, thereby maintaining developmental cycling and viability (Sandoz et al., 2014).

Subsequently, ACCM-D, a further nutritionally defined medium, was developed to facilitate more precise metabolic analysis. Although replication in ACCM-D was slower than in ACCM-2, ACCM-D supported an extended logarithmic phase, improved viability, and higher final yields, reaching approximately 10^9^ GE/mL compared with approximately 10^8^ GE/mL in ACCM-2. Lower GE/FFU and genome equivalent-to-clony-forming unit (GE/CFU) ratios further indicated enhanced viability of ACCM-D grown bacteria (Sandoz et al., 2016).

In parallel, a Vero cell extract-based medium was developed as another host cell-free system. This medium contains small hydrophilic molecules and peptides smaller than 3 kDa, which were proposed to provide essential growth factors for intracellular pathogens. *C. burnetii* was propagated in this medium at 37 °C under microaerophilic conditions supplemented with CO_2_ (Singh et al., 2013).

Overall, the progression from CCM to ACCM, ACCM-2, and ACCM-D has enabled host cell-free propagation of *C. burnetii*. These was achieved by recreating acidic, low-oxygen intracellular environment and supplying amino acids as primary nutrients, thereby supporting growth, developmental transitions, viability, and various downstream experimental applications.

## 8 Concluding remarks

Based on the literature reviewed, Table 1 summarizes the advantages and limitations of the available isolation methods. Figure 1 integrates the major specimen sources with their corresponding isolation and propagation platforms, providing a practical framework for method selection according to specimen characteristics and intended downstream applications.

**TABLE 1:** Comparison of *C. burnetii* isolation and cultivation methods.

Method	Advantages and purpose	Limitations and caveats
Animal inoculation	• Provides selective *in vivo* enrichment of viable organisms from heavily contaminated or low-burden specimens • Maintaining or recovering the virulent phase I phenotype	• Less standardized and requires significant time, cost, and labor • Immunodeficient models may be less effective at excluding microbial contaminants
Embryonated chicken egg inoculation	• Ideal for biological amplification and high-yield production of bacteria for antigen or vaccine preparation • Experimental generation of LPS phase-transition variants	• Requires precise harvest timing to maximize yield • Repeated passage may unintentionally alter the LPS phase • Egg-derived allergens limit use of the antigen in egg-allergenic recipients
Mammalian cell culture	• The standard method for primary isolation and propagation • Essential for studying infectivity, tropism, and host-pathogen interactions	• Phase I strains generally exhibit lower infectivity in cell culture than phase II strains • Success may be dependent on host-cell susceptibility and requires prolonged incubation for low-input samples
Host cell-free culture	• Facilitates pure propagation and expansion without host cells using ACCM-2 or ACCM-D • Useful for metabolic analysis and genetic manipulation	• Growth is strain- or genotype-dependent and not universal for all naturally circulating isolates • Demands strict control of pH, microaerophilic conditions, and medium quality

**FIGURE 1:**
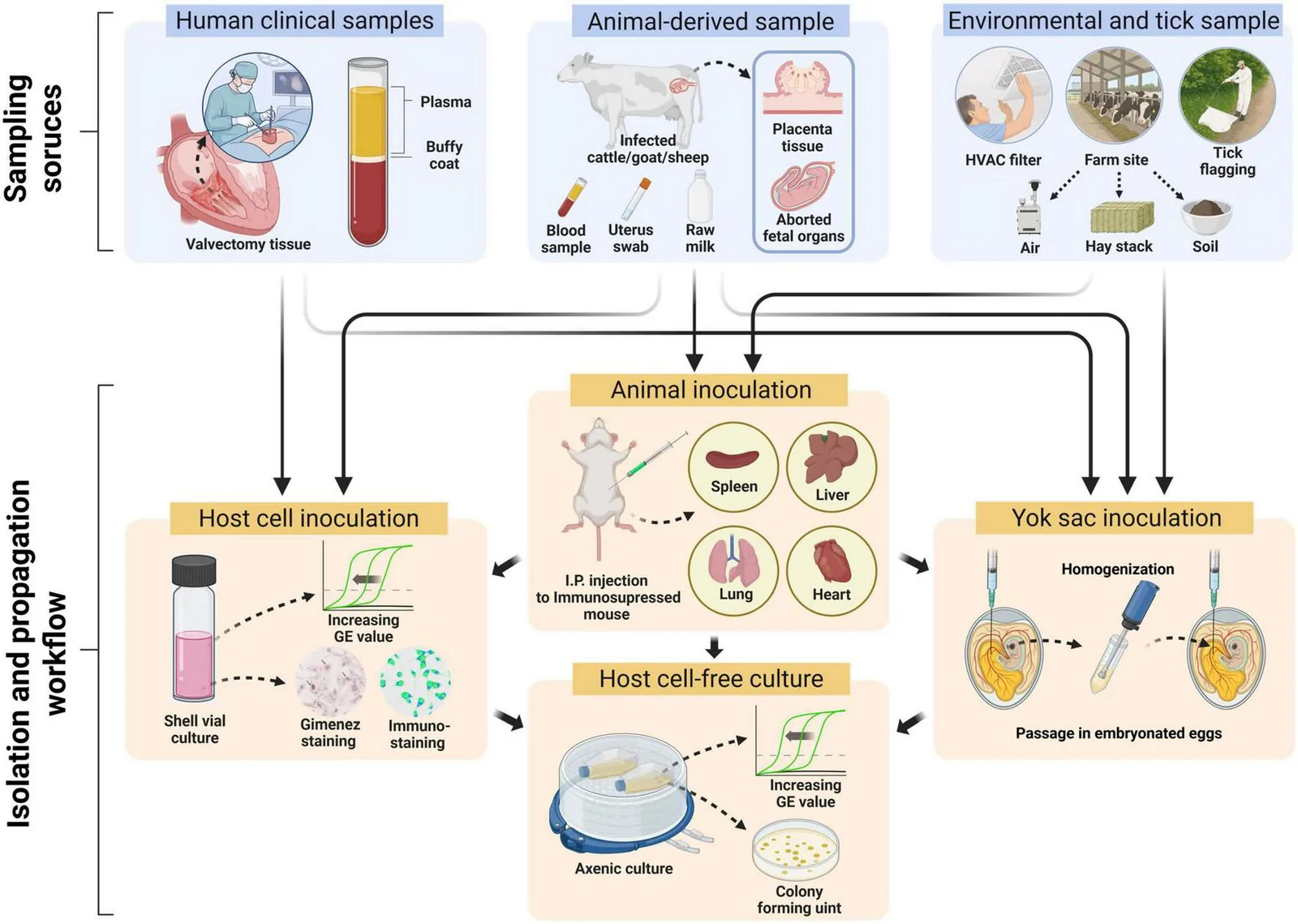
Sampling sources and workflow for the isolation and propagation of *Coxiella burnetii*. Human clinical, animal-derived, environmental, and tick-derived samples are linked to isolation method reported in the reviewed studies, including animal inoculation, mammalian host-cell inoculation, and yolk sac inoculation of embryonated chicken eggs. Animal inoculation may provide *in vivo* enrichment before following isolation workflow, whereas host cell and yolk sac systems support primary isolation and subsequent propagation. Recovered organisms can be further expanded in host cell-free culture, with growth assessed by increases in genome equivalents, staining-based detection, or colony-forming-unit assays. Arrows indicate possible isolation, enrichment, and propagation pathways. GE, genome equivalents; I.P., intraperitoneal.

Despite substantial progress, *C. burnetii* isolation remains challenging due to low recovery rates, platform-dependent performance, and insufficient predictors of culture success. Shell vial culture, while the most widely used and broadly applicable primary isolation method, is not well-suited for high-throughput processing of large sample sets. Therefore, improved pre-culture sample selection is essential.

Since PCR positivity does not always indicate viable organisms, correlations among Ct values, specimen type, storage conditions, bacterial viability, and culture outcome are needed to define practical criteria for prioritizing samples. Interpretation is further complicated because *IS1111* is a multi-copy element, so a given Ct value reflects the number of gene copies rather than the number of organisms. The number of viable organisms available for culture may therefore be considerably lower than the value suggests. Single-copy targets such as *com1* and *htpAB* would give values that are more directly comparable across specimens and laboratories. Viability-associated molecular detection, such as ethidium monoazide- or propidium monazide-based real-time PCR, could help distinguish DNA from viable and nonviable organisms. However, its value for predicting successful *C. burnetii* isolation requires more thorough validation (Mori et al., 2013). Another strategy for improving throughput is using high-content screening platforms, which combine optimized cell culture formats with automated image-based detection of infected cells (Francis et al., 2020). Nevertheless, further optimization using diverse genotypes and strains, along with susceptible or permissive cell types, is necessary to determine whether macrophage-lineage, fibroblast, epithelial, placental, or host species-matched cells are advantageous for specific isolate groups.

Tick-derived samples represent a particularly important area for future refinement. As ticks are increasingly collected through vector-borne disease surveillance programs, they may offer opportunities to broaden the ecological and genetic diversity of *C. burnetii* isolates. However, PCR-positive results should be interpreted cautiously because Coxiella-like endosymbionts are not uncommon in hard and soft ticks (Arthan et al., 2015; Smith et al., 2015; Brenner et al., 2021). Accordingly, isolation attempts from tick-derived samples should be preceded by confirming *IS1111*-positive results using additional *C. burnetii*-informative targets, amplicon sequencing, and, when feasible, phylogenetic or metagenomic analyses to exclude Coxiella-like endosymbionts.

Embryonated egg systems retain value as biological amplification tools, especially for antigen- or vaccine-related applications. Although domestic chickens do not appear to develop overt disease or egg contamination, yolk sac tissues may provide a viable, nutrient-rich, lipid-rich, and endocytically active extraembryonic niche favorable for intracellular replication. Understanding this biological basis may guide improved yolk sac-based production systems or biomimetic host-cell culture models.

Advances in host cell-free axenic media have transformed *C. burnetii* propagation, yet current host cell-free media are not universal isolation tools. For example, Kersh et al. (2016) demonstrated that several genotype groups, including MST1-7/30, MST8, and MST9-10/27-28/31, failed to grow in ACCM-2. These isolates carried the QpRS plasmid, though it remains unclear whether the relevant growth determinants are plasmid- or chromosome-encoded. Therefore, while direct sample inoculation into host cell-free media has been successful in some instances (Omsland et al., 2011; Boden et al., 2015), axenic culture should be considered complementary to other methods as primary inoculation. This genotype-dependent limitation underscores the need for axenic media that support a wider range of naturally circulating *C. burnetii* strains.

In summary, no single platform is optimal for all *C. burnetii* isolation attempts. High-burden, well-preserved clinical or reproductive tissues may be initially directed to shell vial or other susceptible cell culture systems. Heavily contaminated or low-burden specimens may necessitate animal or egg-based enrichment. Host cell-free media are best utilized as complementary propagation systems when strain permissiveness is likely. To enable comparisons of isolation outcomes across laboratories and expand representative *C. burnetii* isolate collections, standardized reporting of specimen matrix, storage conditions, qPCR targets and Ct values, decontamination procedures, culture platform, incubation duration, passage number, and confirmation method will be essential.
